# Scalp pain or tenderness may offer diagnostic clues regarding cicatricial alopecia subtype

**DOI:** 10.1016/j.jdin.2025.03.001

**Published:** 2025-04-04

**Authors:** Noelle Desir, Iain Noel Encarnacion, Temitayo A. Ogunleye, Susan C. Taylor

**Affiliations:** a Weill Cornell Medical College, New York, New York; b Department of Dermatology, Perelman School of Medicine at the University of Pennsylvania, Philadelphia, Pennsylvania; c Eastern Virginia Medical School, Norfolk, Virginia

**Keywords:** alopecia, clinical research, erythema, general dermatology, hair, itch, medical dermatology, pruiritus, scarring alopecia, sign, symptom

*To the Editor:* Cicatricial (scarring) alopecias are a group of rare, chronic conditions that cause permanent hair loss, and symptoms such as itching, tenderness, and dysesthesia may contribute to poorer quality of life among cicatricial alopecia patients.^1^^,^^2^ Scalp symptoms may play a critical role in assessing disease severity, yet there is limited literature on cicatricial alopecia symptomatology.^3^ Early identification and disease-specific treatments are essential for halting disease progression and preventing irreversible follicular damage.^1^^,^^2^ This study investigates variations in scalp symptoms across cicatricial alopecia subtypes, aiming to provide insights that may facilitate more timely diagnosis and effective management of central centrifugal cicatricial alopecia (CCCA), lichen planopilaris (LPP) and frontal fibrosing alopecia (FFA).

We performed a retrospective chart review of cicatricial alopecia patients at the University of Pennsylvania between January 1, 2019 and December 31, 23. Electronic health records were queried by *ICD-10-CM* code for adults with CCCA (L66.8), LPP (L66.1), and FFA (L66.9). Diagnoses were verified using provider documentation. Of 2040 total cicatricial alopecia cases, a representative sample was obtained by randomly analyzing 10% of cases from each subtype. Every unique patient reported scalp signs/symptoms was recorded once per patient, and patients may have reported more than 1 scalp sign/symptom in the study period. Fisher’s exact test analyzed categorical variables.

Of the 204 cicatricial alopecia cases analyzed, 106 were CCCA and 98 were LPP or FFA. Mean age (SD) of CCCA vs LPP/FFA cases were 46.65 years (SD 14.11) vs 59.03 years (SD 13.10), respectively (Table I). CCCA patients were 100% female, whereas LPP/FFA was 93% female and 7% male (Table I). CCCA patients were 93% Black/African American. In contrast, the LPP/FFA group was 70.4% White and 15% Black/African American (Table I). Pain, tenderness, or soreness was reported by 28% CCCA and 13% LPP/FFA patients (*P* < .01, Table II). There was no difference in reports of itch (*P* = .89), or burning, tingling, or another dysesthesia (*P* = .58) between subtypes (Table II). Scalp erythema was reported by 15% LPP/FFA and 2% CCCA patients (*P* < .01, Table II). LPP/FFA vs CCCA patients were more likely to be asymptomatic (24% vs 7%, *P* < .01, Table II). Limitations of this retrospective study are small sample size and missing data.

**Table I:** Demographic characteristics of cicatricial alopecia patients

Demographic characteristics	CCCA (*n* = 106)	LPP/FFA (*n* = 98)	Total (*n* = 204)
Age in y (mean ± SD)	49.65 ± 14.11	59.03 ± 13.10	-
Sex			
Male	0 (0.0%)	7 (7.1%)	7 (3.43%)
Female	106 (100.0%)	91 (92.9%)	197 (95.17%)
Race/Ethnicity			
White	1 (0.9%)	69 (70.4%)	70 (34.3%)
Black/African American	99 (93.4%)	15 (15.3%)	114 (55.88%)
Asian	1 (0.9%)	1 (1.0%)	2 (0.98%)
Native American/American Indian	0 (0.0%)	0 (0.0%)	0 (0.00%)
Hispanic/Latino	0 (0.0%)	1 (1.0%)	1 (0.49%)
Other∗	5 (4.7%)	12 (12.2%)	17 (8.33%)

*CCCA*, Central centrifugal cicatricial alopecia; *LPP/FFA*, lichen planopilaris/frontal fibrosing alopecia.

∗ Other or not documented.

**Table II:** Self-reported scalp signs/symptoms of cicatricial alopecia patients

Reported scalp signs/symptoms∗	CCCA (*n* = 106)	LPP/FFA (*n* = 98)	Total (*n* = 204)	*P* value†
Pain, tenderness, or soreness	30 (28.3%)	13 (13.3%)	43 (21.08%)	**<.01**
Itch	51 (48.1%)	46 (46.9%)	97 (47.55%)	.89
Erythema	2 (1.9%)	15 (15.3%)	17 (8.33%)	**<.01**
Irritation	2 (1.9%)	2 (2.0%)	4 (1.96%)	1
Scale, flaking, or dandruff	5 (4.7%)	7 (7.1%)	12 (5.89%)	.56
Burning, tingling, or another dysesthesia	6 (5.7%)	8 (8.2%)	14 (6.86%)	.58
Bumps or folliculitis	3 (2.8%)	6 (6.1%)	9 (4.41%)	.32
Asymptomatic	7 (6.6%)	23 (23.5%)	30 (14.71%)	**<.01**
Not documented	3 (2.8%)	23 (23.5%)	26 (12.75%)	**<.01**

Bolded values are statistically significant.

*CCCA*, Central centrifugal cicatricial alopecia; *LPP/FFA*, lichen planopilaris/frontal fibrosing alopecia.

∗ Patients may have more than 1 reported scalp symptom.

† Fisher’s exact test analyzed categorical variables.

We found that scalp symptoms are significantly associated with specific cicatricial alopecia subtypes, emphasizing the importance of symptom assessment in clinical evaluations. Dermatologists may wish to query all alopecia patients about the presence and severity of scalp symptoms using a standardized scale (eg, 0 = asymptomatic to 10 = extremely symptomatic) to establish a baseline for monitoring disease activity and treatment response. While erythema is less often associated with CCCA compared to LPP/FFA,^1^ erythema may be underappreciated in darker skin tones underscoring the need for improved diagnostic tools and awareness in diverse populations. Future research should prioritize larger, prospective studies to further clarify symptom patterns, particularly in ambiguous cases (such as indistinguishable CCCA vs LPP).^4^ Integrating symptom-based assessments into clinical practice and therapeutic trials can enhance treatment monitoring, optimize management strategies, and ultimately improve outcomes for patients with cicatricial alopecias.

## Conflicts of interest

Dr Taylor has served as a consultant, advisory board member, and/or speaker for AbbVie, Arcutis, Armis Scientific, Avita, Beiersdorf, Biorez, Bristol-Myers Squibb, Cara Therapeutics, Dior, Eli Lilly, EPI Health, Evolus, Galderma, GloGetter, Hugel America, Incyte, Johnson & Johnson, L’Oreal USA, MedScape, MJH LifeSciences, Pfizer, Piction Health, Sanofi, Scientis US, UCB, and Vichy Laboratories. She has received royalties from McGraw-Hill. She has served as an investigator for Allergan, Concert Pharmaceuticals/Sun Pharma, Croma-Pharma GmbH, Eli Lilly, and Pfizer. Dr Ogunleye has served as an advisory board member for Beiersdorf. Authors Desir and Encarnacion have no conflicts of interest to declare.
